# Dispersal Mutualism Incorporated into Large-Scale, Infrequent Disturbances

**DOI:** 10.1371/journal.pone.0132625

**Published:** 2015-07-07

**Authors:** V. Thomas Parker

**Affiliations:** Department of Biology, San Francisco State University, San Francisco, California, United States of America; Australian National University, AUSTRALIA

## Abstract

Because of their influence on succession and other community interactions, large-scale, infrequent natural disturbances also should play a major role in mutualistic interactions. Using field data and experiments, I test whether mutualisms have been incorporated into large-scale wildfire by whether the outcomes of a mutualism depend on disturbance. In this study a seed dispersal mutualism is shown to depend on infrequent, large-scale disturbances. A dominant shrubland plant (*Arctostaphylos* species) produces seeds that make up a persistent soil seed bank and requires fire to germinate. In post-fire stands, I show that seedlings emerging from rodent caches dominate sites experiencing higher fire intensity. Field experiments show that rodents (*Perimyscus californicus*, *P*. *boylii*) do cache *Arctostaphylos* fruit and bury most seed caches to a sufficient depth to survive a killing heat pulse that a fire might drive into the soil. While the rodent dispersal and caching behavior itself has not changed compared to other habitats, the environmental transformation caused by wildfire converts the caching burial of seed from a dispersal process to a plant fire adaptive trait, and provides the context for stimulating subsequent life history evolution in the plant host.

## Introduction

Disturbances play a critical role in the dynamics of communities [1] and the relationship between disturbance and mutualisms has received considerable attention [2,3]. Current mutualism models concerning disturbance, however, conclude that they disrupt mutualisms by modifying behavior or by reducing species richness or population size [4–7]. Most of this research has investigated pollination, dispersal or protection mutualisms in the context of anthropogenic fragmentation [8–12]. Biological invasions similarly impact ecosystems in a parallel process and results of mutualism breakdown have been found [13,14]. The focus on anthropogenic disturbances is appropriate given global human impacts, however, ecologists also have established the key role that natural disturbances play in community assembly and system functioning. Here I investigate a potential dispersal mutualism in the context of large-scale, infrequent wildfire in California chaparral.

Large-scale, infrequent disturbances such as wind-throws or wildfires have significant impacts in many ecological systems, yet are generally regarded as disrupting interactions among mutualists [7]. How mutualisms may be selected by natural disturbance regimes remains relatively unexplored, even though mutualisms are recognized as critical aspects of ecological evolution [15,16]. This perspective may reflect the assumptions that such disturbances break apart mutualistic interaction networks or that large disturbances are of too great an amplitude or too rare to be incorporated in such networks. Mutualisms are recognized as context-dependent [17–19], yet disturbance regimes also are an ecological context. For example, the role of large-scale disturbances is critical for aspects of community assembly [1,20] and disturbance regimes are incorporated into aspects of an ecosystem’s functioning and dynamics [21–25]. Given this diversity of roles incorporated into an ecosystem’s normal dynamics, a regime of large-scale, extreme and infrequent disturbances similarly are likely incorporated into the framework of mutualisms.

Here I examine a potential dispersal mutualism in the context of wildfire involving rodents that potentially scatter-hoard fruit and the subsequent seed bank responses of four species of *Arctostaphylos* (Ericaceae). *Arctostaphylos* is characterized by dormant seed that produce persistent soil seed banks stimulated by fire [26,27]. The origin of how seeds get buried prior to fire is not well studied, and research focusing on rodents has principally examined their ability to consume seeds [28,29], not whether they might bury seed. Scatter-hoarding rodents, however, would be primary dispersal agents, potentially burying seed and creating the soil seed bank [30]. This caching process may be critical for *Arctostaphylos* seed banks because the fruit and seed are far too large and inappropriately shaped for persistent soil seed banks according to seed bank theory [31]; no physical process can reliably bury these large seed to sufficient depth prior to the return of fire. Because the fire regime involves high intensity canopy fires, the seed must be buried sufficiently deep (2–5 cm) to be able to survive a heat pulse that the fire drives into the soil that kills seed at shallow depths [32].

*Arctostaphylos* also is the most diverse woody genus in western North America [33], in part due to its obligate seeding life history and seed bank responses to fire [34]. The evolution of seed dormancy in *Arctostaphylos* and subsequently an obligate seeding life history both depend on seed surviving fire. The typical fire regimes involving manzanita species involve spatially large-scale fires and fire return intervals shorter than the life expectancy of individuals and so fire can impact population traits by selection [25]. Rodent seed-caching behavior and the environmental conditions produced by fire may have contributed to the evolution of seed dormancy and obligate seeding because together they would create appropriate selective conditions. My questions in this research are 1) do rodents scatter-hoard *Arctostaphylos* fruit, 2) are the fruit buried to sufficient depth to survive fire, and 3) in post-fire stands of *Arctostaphylos*, does evidence exist that scatter-hoarding may be important for seed survival?

## Methods

### Sites

The research approach was to quantify patterns of post-fire seedling establishment as to whether seedlings emerged from apparent rodent caches. To determine if rodents cache fruit and to what depths, fruit were placed out in the field in older chaparral stands and caches recovered and analyzed. Seedling establishment was measured in three chaparral communities during the first spring of post-fire regeneration following three different wildfires in California, USA; Summit Fire (Santa Cruz Mts, May 2008, 37°04’02.03”N, 121°48’07.57”W), Martin Fire (Santa Cruz Mts, June 2008, 37°03’02.45”N, 122°08’22.35”W), and the Cub Complex (June-July 2008, northern Sierra Nevada Mts, Tehama County, 40°11’48.43N, 121°31’19.23”W). The first two fires were accidentally initiated by anthropogenic ignition, but occurred during the typical fire season (May-October) with normal fire behavior and were between 130–150 ha; the Cub Fire Complex was a series of lightning-ignited fires that merged, burning almost 4000 ha. Experiments on rodent caching were performed at the University of California Ft. Ord Natural Reserve near Marina, Monterey County, California (36°40’51.12”N, 121°46’33.43”W). The chaparral at this site is a maritime community on stabilized dunes dominated by two *Arctostaphylos* species, *A*. *tomentosa* and *A*. *pumila*. The chaparral at Ft. Ord had not burned for over 60–80 years at the time of the experiments.

### Assessment of seedlings from caches and fire intensity

Seedling germination was assessed for whether seedlings emerged from rodent caches. The number of seedlings were counted in 20–1 x 2 m plots per site two months following germination (May-July); specific plots were selected using a random number table for x-y coordinates on a 100 x 100 m gridded site. Numbers of seedlings were counted for each of two categories, ‘individual’ or ‘cached’. Seedlings were classified as ‘individual’ if only 1–3 seedlings arose from the same hole, while 4 or more (up to 35 in this study) were classified as arising from a rodent cache. Individual fruit in *Arctostaphylos* normally contain 4–7 endocarps, and of these, only about a third are viable [27]. I made the assumption that 1–3 seedlings would represent a single fruit and be classified as non-cached fruit; however, single fruits can be abiotically buried or cached, so this assumption may underestimate rodent caching. Selecting larger numbers to represent rodent caches represented two or more fruit; in practice classification was not difficult, as most seedlings were either singles or in groups greater than 10. In these post-fire stands, fire intensity was measured using an indirect technique that quantifies stem consumption (fire severity); burned skeletal remains of shrubs were used to quantify the fire intensity experienced in each plot by measuring the diameter of the smallest-sized stems over each plot (minimum stem diameter) [35]. Small diameter stems would represent lower fire severity (intensity) while larger diameters represent higher magnitudes. Because the same plant species stems were measured for each plot, the data were consistent relative measures of intensity within each site [35]. This technique has been successfully used in a number of studies [35–38].

### Rodent caching experiment

To assess whether rodents scatter-hoarded fruit and the depth of rodent caching, fruit at one site was collected from two *Arctostaphylos* species, *A*. *tomentosa* and *A*. *pumila* in August and were placed out in early October within experimental flats. The fluorescent powder technique [39] was used to follow rodent movement to potential caches. Fluorescent powder was thinly spread over the surface of shallow, circular aluminum pans, 35 cm in diameter, that had fine textured sandpaper glued to the bottom. Twenty of these pans were placed beneath shrubs near sunset along a 300 m transect determined by a trail cut through the chaparral. Each site was 10–15 m from the previous site, and pans were placed well off of the main trail. Approximately 60–80 fruit were provided in a petri dish that was glued in the middle of each pan and left overnight. The pans were prepared a week in advance of the field experiment and powder-free disposable gloves were worn when handling the pans and adding the fruit to the petri dishes. At sunrise, fluorescent rodent trails were followed to suspected caches. Caches were carefully excavated and depth and number of fruit determined.

### Statistical Analyses

All data analyses were conducted in R (v.3.0.1; R Development Core Team 2013). The post-fire seedling data and fire intensity data were analyzed in two ways. The statistical model used in both analyses was that the proportion of total seedlings emerging from rodent caches is a function of fire intensity (measured by fire severity as minimum stem diameter in this study). A generalized linear model was used to perform a logistic regression. Data for seedlings emerging from rodent caches were converted to a proportion of the total number of seedlings in each plot. For a parametric regression analysis, to achieve normal distribution in the data, both seedling data (proportions square root arc sine transformed) and stem diameter values (natural log transformed) were transformed prior to statistical analysis. Each site’s data set was analyzed independently.

### Ethics Statement

The field experiments involving rodents were conducted using protocols approved (#A12-08) by the San Francisco State Institutional Animal Care and Use Committee (IACUC).

## Results

**Fig 1:**
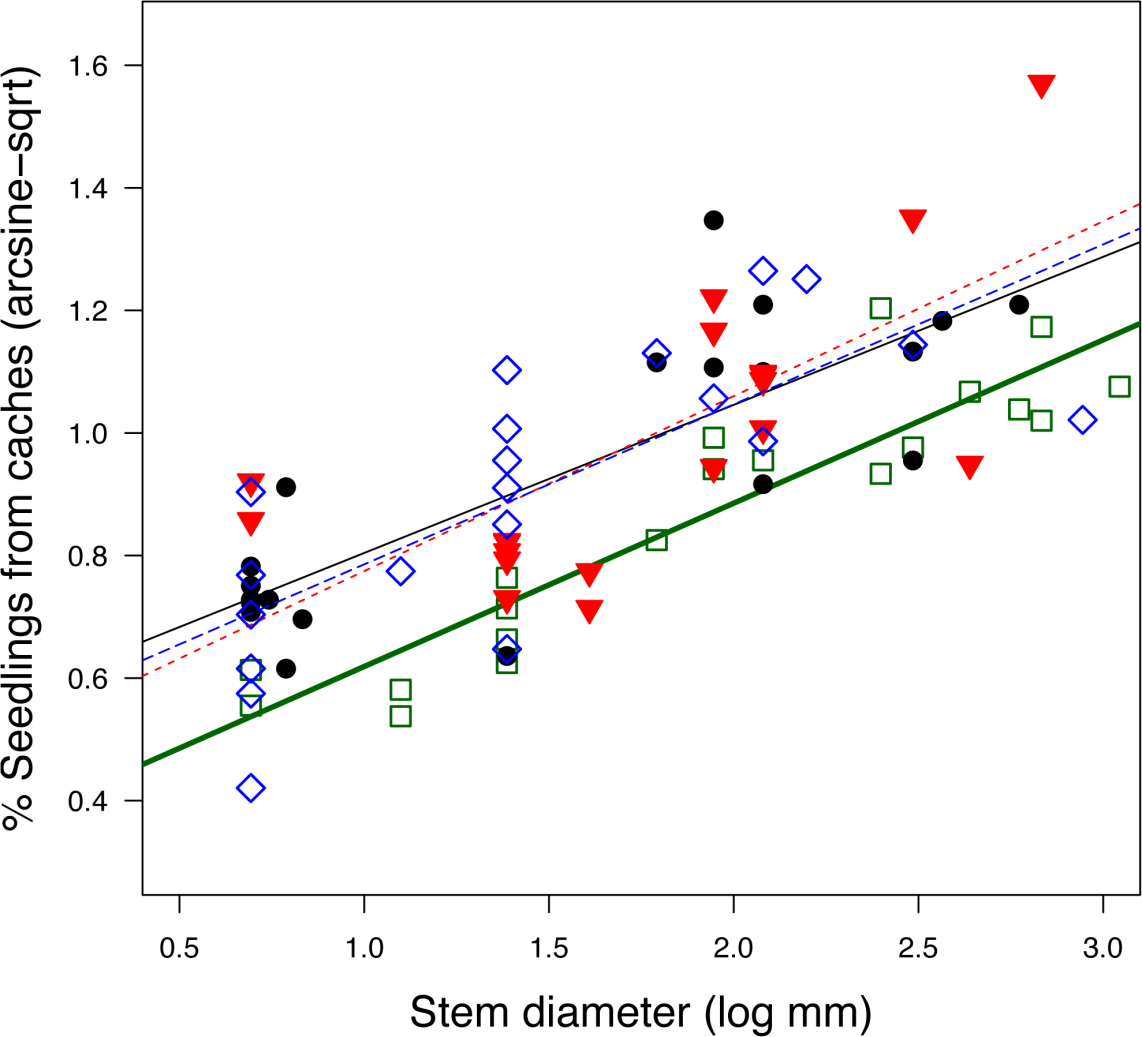
The proportion of seedlings emerging from rodent caches in relation to burned stem diameter (proportional to fire intensity). Seedling data are square-root arc-sin transformed and represent the percentage of seedlings in a plot that emerge from rodent caches. The fire intensity data are natural log transformed and represent smallest stem diameter of the resident burned *Arctostaphylos* species above the plot. Species represented in this plot include *A*. *sensitiva* (solid squares, y = 0.27x +0.36; F_1,18_ = 102.5, p <0.0001; adj R^2^ = 0.84), *A*. *andersonii* (open triangles, y = 0.29x + 0.49; F_1,18_ = 17.89, p<0.001; adj R^2^ = 0.47), *A*. *silvicola* (solid circles, y = 0.24x +0.56; F_1,18_ = 36.76, p<0.0001; adj R^2^ = 0.65), and *A*. *patula* (open diamonds, y = 0.26x +0.52; F_1,18_ = 24.03, p<0.001; adj R^2^ = 0.55).

In post-fire analysis of seedling emergence in recent fires, I found effective *Arctostaphylos* seed banks comprised at least 40–60% of seed buried by rodents in caches and the rest by unknown processes. This estimate is based on the average of plots at the lowest fire intensity and assuming isolated seedlings arise from seed not buried by rodents, which underestimates caching. At sites with higher fire intensity, fewer seedlings emerged from the soil, an expected result based on a deeper killing heat pulse. But as fire intensity increases, seedlings arising from rodent caches become the dominant source of survivors (Fig 1). At higher fire intensities, almost all the seedlings originated from caches, a pattern found for four species sampled that were part of three separate fires.

The results of the generalized linear model conducting logistic regression had very low p values for all four species, 0.001 (*A*. *andersonii*), 1e^-09^, (*A*. *patula*), and 1e^-16^ (*A*. *sensitiva*, *A*. *silvicola*); in all cases the residual deviance was reduced considerably (10.0–101.5 on 18 df). Similarly, for linear regressions performed on transformed data, statistical results are represented by the following p-values: *A*. *sensitiva* (y = 0.27x +0.36; F_1,18_ = 102.5, p <0.0001; adj R^2^ = 0.84), *A*. *andersonii* (y = 0.29x + 0.49; F_1,18_ = 17.89, p<0.001; adj R^2^ = 0.47), *A*. *silvicola* (y = 0.24x +0.56; F_1,18_ = 36.76, p<0.0001; adj R^2^ = 0.65), and *A*. *patula* (y = 0.26x +0.52; F_1,18_ = 24.03, p<0.001; adj R^2^ = 0.55).

**Fig 2:**
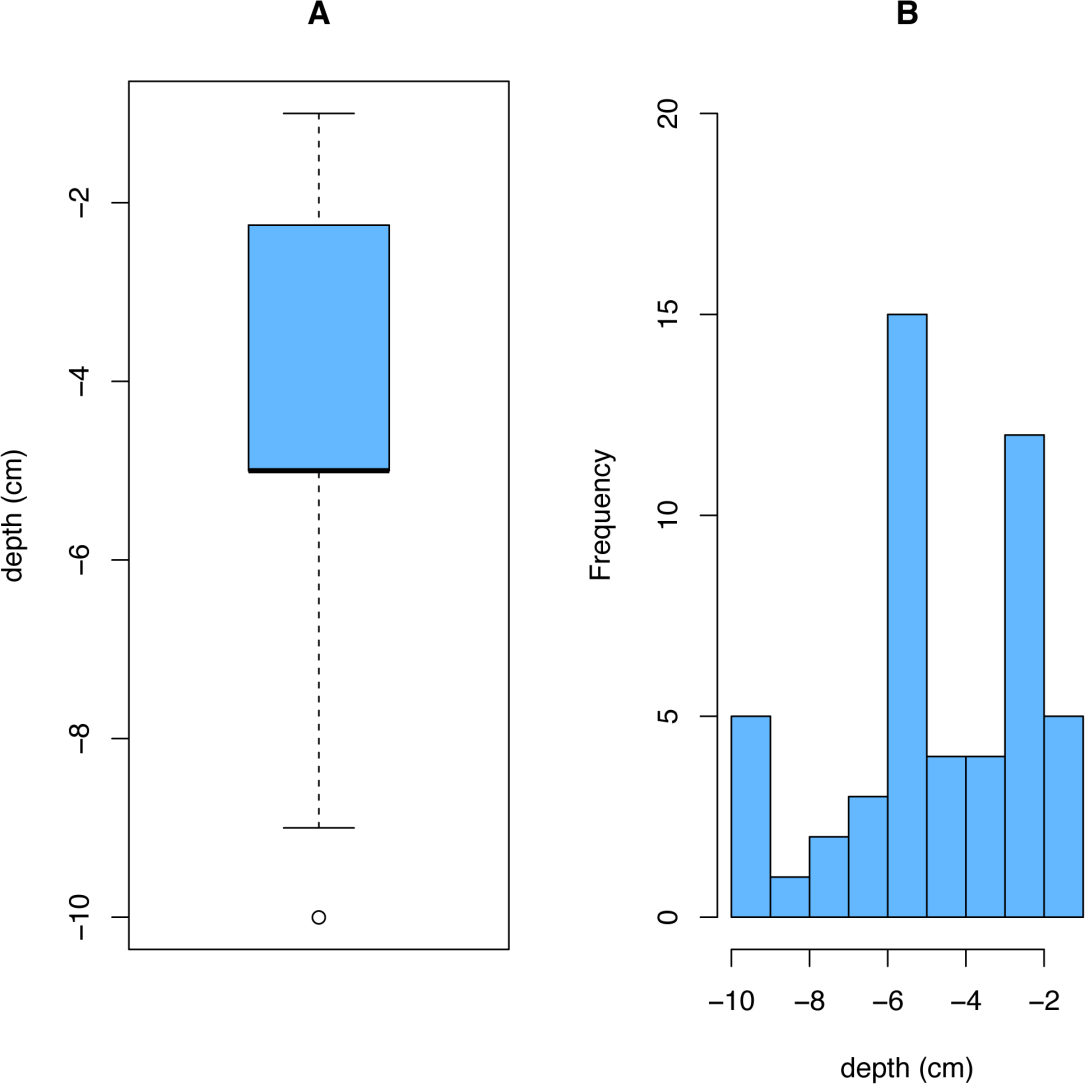
Depth distribution of rodent caches. Data represent the top of the seed/fruit caches in the field experiment as excavated. (A) The average depth was 4.4 + 0.35 cm (mean and standard error; dark line shows median), while (B) the histogram illustrates the range and distribution of cache depths.

**Fig 3:**
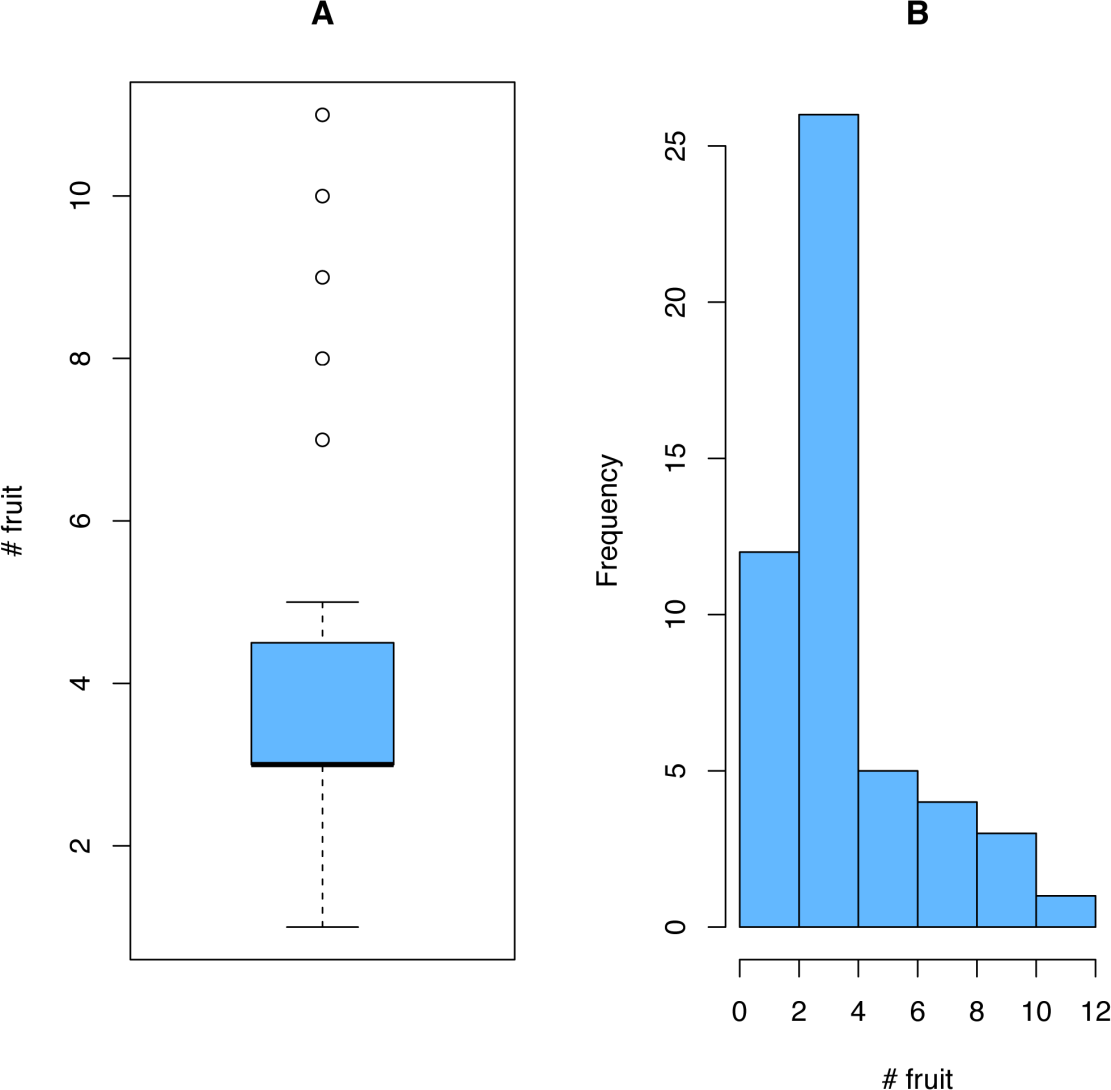
Number of fruit stored in caches. (A) The average number of fruit per cache was slightly over 4 fruit (4.1 + 0.32 fruit [mean and standard error]; dark line shows median). (B) The histogram illustrates the range and distribution of fruit number per cache. *Arctostaphylos* fruit range in seed number and viability, but average between 3 and 7 viable seed per fruit as a way of assessing potential cache size.

In the fruit provision experiment, fifty-one caches were found, but two were located in deep litter and they were omitted from calculations. Fruit were buried to an average of 4.4 + 0.35 cm (mean and S.E.) (Fig 2A). While most seed were near the surface, the depth range of burial was large and a few fruit were as deep as 10 cm (Fig 2B). The average cache for this experiment was a little over four fruit per cache (4.1 + 0.32 S.E.), which means an average of 12–30 viable seeds (Fig 3).

## Discussion

Mutualistic interactions can also incorporate large-scale, infrequent disturbance regimes as indicated by these results. The strength and importance of mutualisms is clearly dependent on environmental context and this study demonstrates that rodent dispersal and caching behavior itself may be similar to scatter-hoarding in other habitats, but the shift in abiotic conditions resulting from disturbance changes the nature or impact of the interaction. Fire regimes would select for scatter-hoarding burial as an insurance against extreme fire events that otherwise would eliminate the population from a site. Insurance against failure is critical for these species with persistent seed banks, and especially for obligate seeders, whose adults are killed by the fire. This process is analogous to models developed for desert annuals [40,41], in which persistence provides the insurance against extreme drought events. Caching by scatter-hoarders permits seed survival of fire, but such caching does not involve any change in behavior of the rodents or any change in the production of seed by the host plant.

Scatter-hoarding is an important process for a number of species, but studies have focused principally on plants with transient seed banks and examined aspects such as masting, spatial distances to avoid conflicts with parents or siblings, gene flow or other aspects of population ecology [30]. Theory on seed dispersing mutualists has included ant dispersal [42] and dispersal by birds or mammals [43] in the context of dispersal to safe sites that improve establishment. While a fire burns above-ground biomass, belowground a heat pulse through the soil near the soil surface may exceed the tolerance of buried seed, creating a kill zone near the surface. The kill zone is thought to be down to 2–5 cm in depth depending on fire intensity [32,36]. In this circumstance, the fire regime would select for mutualists that can disperse seed to safe sites, which is a depth below the heat pulse kill zone. Directed dispersal to safe sites in canopy-fire vegetation could be characterized as spatially vertical placement into the soil below killing temperatures generated by fire rather than by horizontal movement as it is usually considered. Wildfire is an extreme, but common abiotic process in many ecosystems. Fire intensity also varies considerably among and within individual fires and is a major source of site heterogeneity after fire [25]. Adaptive plant responses in the context of such strong abiotic processes include a range of traits [25] and should also influence mutualisms, such as the scatter-hoarding of seed and subsequent development of soil seed banks.

From the perspective of the plant populations these results indicate that a seed dispersal mutualism can be a considerably strong positive interaction in the context of wildfire. This process results in a functional fire adaptation for the plant population. Bronstein [44] predicted that selection should be stronger on facilitated species than on the facilitators. In this case, the results provide correlated support for Bronstein’s hypothesis. *Arctostaphylos* has two characteristics not found in close relatives, traits that currently rely on this mutualism. One character is seed dormancy stimulated by fire and the development of persistent soil seed banks. Given the evolutionary trend in the subfamily Arbutoideae from soft bird-dispersed fruit in *Arbutus* to dry fruit with seeds encased in hard nut-like endocarps in *Arctostaphylos*, scatter-hoarding burial would support an evolutionary model of selection by regimes of drought, wildfire and seed predation that would lead to the development of seed dormancy in *Arctostaphylos*. *Arctostaphylos* also is the only genus in the subfamily subsequently to evolve species with an obligate seeding life history, a character that probably led to the greater diversity in this genus [33,34]. Bronstein [44] also suggested that evolutionary changes should demonstrate a geographic mosaic of interaction outcomes; *Arctostaphylos* does have a globally widespread species, *A*. *uva-ursi*, on which this idea could be tested, as it lives in a diversity of habitats such as coastal sand dunes, boreal forests, and vegetation with wildfire such as the pine barrens of eastern U.S. along the coast from New Jersey to Massachusetts.

Mutualism research has created a number of categories as a framework for investigating mutualistic interactions, such as resource exchanges, protection, directed dispersal and others. This seed dispersal mutualism simultaneously fits several categories. Scatter-hoarding rodents act as effective directed dispersal agents, but by burying seed vertically into the soil. Additionally, because the heat pulse of fire can kill seeds close to the surface, performing as an abiotic seed predator [45], caching also simultaneously acts as a protection mutualism, especially for obligate seeders. While anthropogenic disturbances may disrupt mutualistic interactions and cause interaction networks to degrade [8–12], this is likely due to those disturbances being outside the normal range of disturbance regimes experienced by the ecosystem. In contrast, in this example mutualistic outcomes depend on the natural, large-scale, infrequent disturbance regime. This suggests that natural disturbance regimes would have differential facilitory impacts on other mutualistic interactions as well. As more diverse examples of mutualisms are explored, their incorporation into community and evolutionary theory will also expand the complexity and dimensionality of mutualistic interactions.

## Supporting Information

S1 Table Numbers of seedlings emerging from rodent caches (cached), total number of seedlings in the plot (total) and the minimum stem diameter above the plot (mm stem diam.), for four different *Arctostaphylos* species, *A*. *sensitiva*, *A*. *silvicola*, *A*. *andersonii* and A. patula. Data for each species from different plots and sites.
(DOCX)

S2 Table Depth and number of *Arctostaphylos* fruits found in rodent caches during the field fruit provisioning experiment. (DOCX)
