# Supplementary material for: Dispersal Mutualism Incorporated into Large-Scale, Infrequent Disturbances
Source: PLoS One. 2015 Jul 7;10(7):e0132625. doi: 10.1371/journal.pone.0132625 (PMC4495039; doi:10.1371/journal.pone.0132625)
Supplement: S1 Table — Data for each species from different plots and sites. (DOCX) [file pone.0132625.s001.docx]

S1 Table Supporting Information. Numbers of seedlings emerging from rodent caches (cached), total number of seedlings in the plot (total) and the minimum stem diameter above the plot (mm stem diam.), for four different *Arctostaphylos* species, *A. sensitiva*, *A. silvicola, A. andersonii* and *A. patula*. Data for each species from different plots and sites.

| # *A. sensitiva*  cached | # *A. sensitiva*  total | mm stem diam. | # *A. silvicola*  cached | # *A. silvicola*  total | mm stem diam. | # *A. andersonii*  cached | # *A. andersonii*  total | mm stem diam. | # *A. patula*  cached | # *A. patula*  total | mm stem diam. |
| --- | --- | --- | --- | --- | --- | --- | --- | --- | --- | --- | --- |
| 16 | 61 | 3 | 18 | 54 | 2.2 | 172 | 301 | 2 | 18 | 54 | 2 |
| 68 | 97 | 7 | 89 | 201 | 2 | 81 | 189 | 5 | 14 | 29 | 2 |
| 25 | 83 | 3 | 33 | 78 | 2 | 145 | 229 | 2 | 13 | 44 | 2 |
| 57 | 133 | 4 | 36 | 102 | 4 | 63 | 129 | 5 | 3 | 18 | 2 |
| 56 | 73 | 14 | 58 | 131 | 2.1 | 52 | 117 | 4 | 29 | 47 | 2 |
| 40 | 117 | 4 | 77 | 155 | 2 | 68 | 127 | 4 | 18 | 43 | 2 |
| 40 | 144 | 2 | 51 | 124 | 2.3 | 43 | 80 | 4 | 44 | 90 | 3 |
| 52 | 157 | 2 | 100 | 160 | 2.2 | 34 | 67 | 4 | 58 | 93 | 4 |
| 70 | 102 | 12 | 84 | 192 | 2 | 25 | 48 | 4 | 18 | 27 | 4 |
| 50 | 132 | 4 | 121 | 260 | 2 | 40 | 61 | 7 | 13 | 23 | 4 |
| 34 | 63 | 6 | 84 | 96 | 16 | 72 | 92 | 8 | 24 | 66 | 4 |
| 32 | 49 | 7 | 30 | 45 | 12 | 37 | 56 | 14 | 10 | 14 | 4 |
| 61 | 84 | 17 | 18 | 21 | 13 | 61 | 77 | 8 | 43 | 54 | 4 |
| 62 | 96 | 11 | 17 | 27 | 8 | 60 | 71 | 7 | 9 | 11 | 6 |
| 72 | 97 | 16 | 42 | 48 | 8 | 25 | 35 | 8 | 72 | 95 | 7 |
| 51 | 60 | 17 | 58 | 61 | 7 | 15 | 19 | 8 | 16 | 23 | 8 |
| 24 | 31 | 21 | 75 | 93 | 6 | 15 | 17 | 7 | 10 | 11 | 8 |
| 55 | 115 | 4 | 12 | 15 | 7 | 15 | 17 | 7 | 73 | 81 | 9 |
| 108 | 124 | 11 | 81 | 102 | 8 | 20 | 21 | 12 | 29 | 35 | 12 |
| 60 | 90 | 8 | 73 | 89 | 12 | 13 | 13 | 17 | 24 | 33 | 19 |
