# Supplementary material for: Dispersal Mutualism Incorporated into Large-Scale, Infrequent Disturbances
Source: PLoS One. 2015 Jul 7;10(7):e0132625. doi: 10.1371/journal.pone.0132625 (PMC4495039; doi:10.1371/journal.pone.0132625)
Supplement: S2 Table — (DOCX) [file pone.0132625.s002.docx]

S2 Table Supporting Information. Depth and number of *Arctostaphylos* fruits found in rodent caches during the field fruit provisioning experiment.

| Cache # | depth (cm) | # fruits |
| --- | --- | --- |
| 1 | -4 | 3 |
| 2 | -2 | 3 |
| 3 | -2 | 3 |
| 4 | -6 | 5 |
| 5 | -5 | 1 |
| 6 | -2 | 7 |
| 7 | -1 | 7 |
| 8 | -3 | 2 |
| 9 | -1 | 4 |
| 10 | -5 | 4 |
| 11 | -5 | 2 |
| 12 | -10 | 11 |
| 13 | -7 | 2 |
| 14 | -5 | 9 |
| 15 | -7.5 | 8 |
| 16 | -5 | 1 |
| 17 | -5 | 3 |
| 18 | -3.5 | 5 |
| 19 | -5 | 3 |
| 20 | -5 | 2 |
| 21 | -10 | 3 |
| 22 | -2.5 | 3 |
| 23 | -5 | 5 |
| 24 | -1 | 3 |
| 25 | -5 | 4 |
| 26 | -2 | 4 |
| 27 | -5 | 4 |
| 28 | -9 | 4 |
| 29 | -5 | 3 |
| 30 | -3 | 2 |
| 31 | -2 | 1 |
| 32 | -1 | 3 |
| 33 | -2 | 5 |
| 34 | -3.5 | 3 |
| 35 | -6 | 9 |
| 36 | -2.5 | 4 |
| 37 | -4 | 2 |
| 38 | -2.5 | 4 |
| 39 | -5 | 10 |
| 40 | -2.5 | 3 |
| 41 | -8 | 4 |
| 42 | -1 | 2 |
| 43 | -5 | 5 |
| 44 | -4 | 4 |
| 45 | -4 | 4 |
| 46 | -9 | 4 |
| 47 | -5 | 2 |
| 48 | -10 | 2 |
| 49 | -2 | 7 |
